# Walk the Line: How Successful Are Efforts to Maintain Monogamy in Intimate Relationships?

**DOI:** 10.1007/s10508-018-1376-3

**Published:** 2019-06-18

**Authors:** Brenda H. Lee, Lucia F. O’Sullivan

**Affiliations:** Department of Psychology, University of New Brunswick, Fredericton, NB E3B5A3 Canada

**Keywords:** Monogamy, Infidelity, Relationship maintenance, Investment Model

## Abstract

Monogamy, typically defined as sexual and romantic exclusivity to one partner, is a near-universal expectation in committed intimate relationships in Western societies. Attractive alternative partners are a common threat to monogamous relationships. However, little is known about how individuals strive to protect their relationships from tempting alternatives, particularly those embedded in one’s social network. The current exploratory study was guided by the Investment Model, which states that satisfaction, investments, and perceived alternatives to a relationship predict commitment, which in turn predicts relationship longevity. The study aimed to identify relationship and extradyadic attraction characteristics associated with monogamy maintenance efforts, specifically relationship commitment, as predicted by the Investment Model. The efficacy of monogamy maintenance efforts was assessed via sexual and emotional infidelity measures at a 2-month follow-up. U.S. adults in heterosexual intimate relationships (*N* = 287; 50.2% male; *M* age = 34.5 years; *M* relationship length = 87 months) were recruited online to complete the survey study. Through structural equation modelling, the Investment Model structure was replicated, and relationship commitment predicted use of relationship-enhancing efforts as well as self-monitoring/derogation efforts. Individuals who experienced reciprocated attraction used significantly more avoidance and self-monitoring/derogation efforts than did those who experienced unreciprocated attraction. Ultimately, monogamy maintenance efforts did not significantly predict success in maintaining monogamy at follow-up. These findings have important research, educational, and clinical implications relating to relationship longevity.

## Introduction

Monogamy is the standard adopted by the majority of those in committed romantic relationships in Western societies. It is a relationship form that is viewed as optimal and conferred with many social, financial, and legal benefits (Anderson, 2010; Conley, Ziegler, Moors, Matsick, & Valentine, 2012; Shackelford & Buss, 1997; Ziegler, Conley, Moors, Matsick, & Rubin, 2015). Monogamy can be defined as sexual and emotional exclusivity to one romantic partner (Conley, Moors, Matsick, & Ziegler, 2013). The vast majority of U.S. heterosexual adults in committed romantic relationships endorse sexual exclusivity as the norm; one study found that 99% of married and 94% of cohabiting heterosexual respondents expected sexual exclusivity for themselves and their partners (Treas & Giesen, 2000). Although similar figures have not been established for emotional exclusivity, emotional betrayal and extradyadic intimate behaviors are commonly considered to be monogamy violations (Thompson & O’Sullivan, 2016a; Weiser, Lalasz, Weigel, & Evans, 2014).

A number of societal changes have challenged the practice of monogamy, specifically changes that expose individuals to a range of attractive potential partners. These include high rates of both women and men in the paid workforce (Finkel, Hui, Carswell, & Larson, 2014), longer hours spent at work (Blow & Hartnett, 2005; Treas & Giesen, 2000), increased mobilization of workforces, fragmentation of extended family units, and lower rates of marriage (Blow & Hartnett, 2005; Treas & Giesen, 2000). In particular, greater financial independence (Finkel et al., 2014) and greater proportion of one’s day spent at work and outside of the home compared to previous generations is believed to lower interdependence between partners (Blow & Hartnett, 2005), and increased opportunities to engage in extradyadic relationships (Blow & Hartnett, 2005; Blumstein & Schwartz, 1983; Weaver, 2007).

In addition, there is growing evidence of shifts in Western societal attitudes toward greater permissiveness in relationships and sexuality over the prior few decades, as noted in public attitudes toward premarital sex (Kraaykamp, 2002; Sprecher, Treger, & Sakaluk, 2013), casual sex (Kraaykamp, 2002; Petersen & Hyde, 2010; Sprecher et al., 2013), and extramarital sex (Kraaykamp, 2002; Petersen & Hyde, 2010). These shifts indicate growing tolerance for alternative relationship structures, perhaps spurred in part by the rapid uptake of new forms of social media and other technology (Dewing, 2010; Finkel et al., 2014).

Within intimate relationships, individuals are increasingly relying on their partners to serve needs beyond sexual gratification and emotional support, such as higher-level needs for autonomy, esteem, and self-actualization (Finkel et al., 2014). However, individuals’ investments in time and resources in intimate relationships have not corresponded with the increasing levels of these higher-level demands (Finkel et al., 2014). Decreases in both relationship (Agnew & VanderDrift, 2015; Dainton, 2000; Ogolsky & Bowers, 2013) and sexual satisfaction (Klusmann, 2002; McNulty, Wenner, & Fisher, 2016; Sprecher, 2002) are likely to result from this discrepancy in relationship investments and demands, making monogamy an ideal that is increasingly difficult to maintain.

Monogamy remains the cultural norm despite increasing challenges toward this relationship ideal, and the majority of individuals in relationships aim to be sexually and romantically exclusive. Yet, consistent with these sociocultural shifts, rates of infidelity are high. Approximately half of college-aged individuals (46.8%) reported lifetime infidelity (Thompson & O’Sullivan, 2016b), and approximately one-fifth of individuals (23% of men and 19% of women) reported sexual infidelity in their current romantic relationships (Mark, Janssen, & Milhausen, 2011). Infidelity is most commonly understood as the participation in sexual acts with partner(s) outside of one’s committed romantic relationship where an agreement to maintain sexual exclusivity is in place (Hackathorn, Mattingly, Clark, & Mattingly, 2011). Relationship commitment and quality are consistently and strongly linked with violations of monogamy norms. Relationships characterized with lower commitment (Feldman & Cauffman, 1999; Maddox Shaw, Rhoades, Allen, Stanley, & Markman, 2013; Mark et al., 2011; Whisman, Gordon, & Chatav, 2007), and relationships with low sexual and relationship compatibility are associated with greater rates of infidelity (Mark et al., 2011). Infidelity and its concomitant betrayal of trust are often associated with a subsequent decrease in relationship commitment and with a much greater likelihood for marital or relationship breakup (Allen & Atkins, 2012; Amato & Previti, 2003; DeMaris, 2013). Drawing from the infidelity literature has limited utility for the study of monogamy, however. The ways in which individuals successfully negotiate exclusivity within their intimate relationships in the face of personal, societal, and cultural challenges remains relatively unexplored. What are needed are insights into how monogamy is maintained over time in intimate relationships.

### Defining and Negotiating Monogamy

A small but growing field of research on monogamy has started to reconcile divergent definitions of monogamy (Conley et al., 2012; Ziegler et al., 2015), to examine monogamy as a harm reduction strategy (Britton et al., 1998; Hearn, O’Sullivan, El-Bassel, & Gilbert, 2005; Warren, Harvey, & Agnew, 2012), and to explore the perceptions of consensual non-monogamy in contrast to monogamy (Conley et al., 2012, 2013). Monogamy is typically viewed positively and appears to be multifaceted. Monogamy has been conceptualized to comprise sexual and emotional exclusivity, whether extradyadic attraction is normalized, and the outward appearance of monogamy in one’s relationship (Anderson, 2010). Monogamous relationships are viewed considerably more favorably than non-monogamous relationships and are perceived to be safer, more moral, committed, meaningful, passionate, and trusting than are consensual non-monogamous relationships (Conley et al., 2013). These findings emerge despite consensual non-monogamous individuals demonstrating comparable levels of psychological well-being, relationship adjustment and commitment, jealousy, and sexual satisfaction as monogamous individuals (Rubel & Bogaert, 2015).

Yet monogamy is poorly defined within individual relationships, with most individuals relying on implicit assumptions and not explicit communications about relationship boundaries. Approximately half of individuals (49.7%) in dating relationships have not discussed expectations for monogamy directly or what monogamy constitutes for their relationships (Gibson, Thompson, & O’Sullivan, 2016; Watkins & Boon, 2016). Partners often disagree on which behaviors comprise monogamy within a relationship (Boekhout, Hendrick, & Hendrick, 2003). Furthermore, it is unclear whether explicit negotiations of monogamy expectations result in mutually understood and maintained relationship boundaries, and whether this understanding is in turn associated with monogamy maintenance.

### The Investment Model

The Investment Model (Rusbult, 1980, 1983; Rusbult, Martz, & Agnew, 1998), an extension of interdependence theory (Thibaut & Kelley, 1959), has been used to predict and explain infidelity in dating relationships (Drigotas, Safstrom, & Gentilia, 1999). The Investment Model posits that commitment—the desire and motivation to continue a relationship—is the primary determinant of relationship longevity or termination, with relationship satisfaction, investments, and the perceived quality of alternatives to the relationship predicting levels of relationship commitment (Rusbult, 1980, 1983; Rusbult et al., 1998). Relationship satisfaction is the balance of positive and negative outcomes received from one’s relationship. Investments are tangible and intangible investments into a relationship that one would lose should the relationship end. Perceived quality of alternatives is what one expects to be able to derive from alternative relationship arrangements should the current relationship end. Satisfaction and investment are positively predictive of commitment, whereas perceived quality of alternatives is negatively predictive of commitment (Le & Agnew, 2003). Investment Model variables have been associated with extradyadic romantic and sexual involvement in dating relationships (Drigotas et al., 1999; Martins et al., 2016), and extradyadic involvement was associated with decreased relationship commitment in turn (Drigotas et al., 1999). The Investment Model may, in turn, provide a useful framework for predicting efforts to maintain monogamy in intimate relationships.

### Derogation of Alternatives and Monogamy Maintenance

The derogation of alternatives involves cognitive and perceptual biases that serve to actively minimize the perceived attractiveness of opposite sex alternatives, in service of one’s primary relationship (Ritter, Karremans, & van Schie, 2010). Compared to single individuals, individuals in committed relationships demonstrate negatively biased memory recall of attractive faces (Karremans, Dotsch, & Corneille, 2011), inattention toward attractive opposite sex targets (Maner, Gailliot, & Miller, 2009), automatic self-protective responses toward attractive targets (Plant, Kunstman, & Maner, 2010), and lower ratings of attractiveness toward attractive targets (Johnson & Rusbult, 1989; O’Sullivan & Vannier, 2013).

The derogation of attractive alternatives varies given the level of relationship threat posed by the alternative and by an individual’s level of commitment to their primary relationship (Lydon, Fitzsimons, & Naidoo, 2003; Lydon, Meana, Sepinwall, Richards, & Mayman, 1999). Individuals in highly committed relationships who were informed that an attractive individual showed interest in dating them displayed the strongest derogation of alternatives, demonstrated via lower ratings of the alternative’s attractiveness (Lydon et al., 1999). These findings indicate that reciprocated attraction by an individual to whom one is attracted likely represents higher relationship threat.

However, missing from the research literature is information addressing a broader scope of efforts that individuals employ when facing non-fleeting, attractive alternatives to their relationships. The literature has only explored relatively automatic protective responses against fleeting temptations of extradyadic involvement (Johnson & Rusbult, 1989; Karremans et al., 2011; Lydon et al., 1999, 2003; O’Sullivan & Vannier, 2013; Maner et al., 2009; Plant et al., 2010; Ritter et al., 2010). Information regarding how individuals deliberately respond to potentially more long-standing temptations posing a threat to the maintenance of monogamy is important for generating insights into relationship longevity.

### The Current Study

This research examined monogamy maintenance in terms of efforts that individuals employ when facing an episode of extradyadic attraction. Three main purposes guided the current study: (1) to investigate the role of relationship commitment in influencing how individuals manage their extradyadic attraction; (2) to identify the efficacy of these efforts; and (3) to examine whether differing levels of threat posed by reciprocated and unreciprocated attraction influence levels of monogamy maintenance.

Although this is an exploratory study of monogamy maintenance, we made a number of directional hypotheses consistent with the Investment Model and prior research. We expected that:

#### **H1a**

Relationship satisfaction, investments, and perceived alternatives would predict degree of commitment, replicating the Investment Model structure;

#### **H1b**

Relationship commitment would be positively associated with the range of monogamy maintenance efforts used;

#### **H2a**

The range of monogamy maintenance strategy use at Time 1 would be negatively associated with sexual and romantic infidelity at a 2-month follow-up;

#### **H2b**

Infidelity would be negatively associated with commitment at follow-up; and

#### **H3**

Extradyadic attraction that is reciprocated by an attractive other would represent higher levels of relationship threat and prompt a greater range of monogamy maintenance efforts as compared to unreciprocated extradyadic attraction.

Group differences in gender, relationship status, and explicit monogamy agreements also were examined to explore their relationships to monogamy maintenance strategy use prior to analyses. Gender had been identified as a pertinent factor that influences infidelity, including rates of participation, desire for extradyadic sex, and permissive attitudes toward infidelity (Blow & Hartnett, 2005). Relationship status (e.g., married, cohabiting, or dating) reflects structural commitment (Lydon et al., 1999), beyond measures of attitudinal commitment. Lastly, whether a couple has an explicit monogamy agreement in place may serve to clarify expectations and intentions between partners and aid in monogamy maintenance.

## Method

### Participants

Heterosexual U.S. adults were recruited to complete an anonymous online survey for monetary compensation via Amazon^®^’s Mechanical Turk^®^ (MTurk^®^), a crowdsourcing Web site that allows individuals to complete online jobs of their choosing for monetary compensation (Mason & Suri, 2012). Participants were recruited via advertisements posted on MTurk^®^. Samples recruited via MTurk^®^ are generally more heterogeneous than community, student, or traditionally recruited online samples (Buhrmester, Kwang, & Gosling, 2011; Casler, Bickel, & Hackett, 2013). Eligibility criteria included being involved in a committed, monogamous male–female romantic relationship. Heterosexual participants were chosen for the sample as differences exist in attitudes toward and expectations for monogamy between heterosexual, gay/lesbian, and bisexual individuals in intimate relationships (Hoff & Beougher, 2008; Hosking, 2013; Mark, Rosenkrantz, & Kerner, 2014). The final sample (*N* = 287) consisted of 143 women and 144 men with a mean age of 34.5 years (*SD* = 9.6, range 19–68). Participants identified primarily as White (77.0%), Asian/Pacific Islander (8.0%), and Black (6.6%). Half of the participants (54.4%) indicated that they were in a married or cohabiting relationship; another 45.6% indicated that they were in a dating relationship. The mean relationship length was 87.0 months (*SD* = 94.0, range = 0–481). Participant characteristics are shown in Table 1.

**Table 1:** Descriptive statistics for initial and follow-up samples

Characteristic	Initial recruitment (*n* = 287)	Two-month follow-up (*n* = 131)
Gender		
Female	143	68
Male	144	63
Age in years (SD)	34.5 (9.6)	35.3 (9.9)
Ethnic/racial identification		
White	77%	79.4%
Asian/Pacific Islander	8%	4.6%
Black	6.6%	7.6%
Other	8.4%	8.4%
Relationship status		
Married/cohabitating	54.4%	60.3%
Dating	45.6%	39.7%
Relationship length in months (SD)	87 (94.0)	102.2 (107.5)
Extradyadic attraction		
Yes	61.7%	51.1%^a^
No	38.3%	48.9%^a^
Infidelity in current relationship	12.9%	15.2%
Romantic infidelity	8.4%	11.5%
Sexual infidelity	8%	9.2%
Both	3.5%	5.3%
Investment Model		
Satisfaction^b^	7.3 (1.6)	7.3 (1.7)
Investment^b^	7.4 (1.4)	7.5 (1.2)
Perceived quality of alternatives^b^	3.8 (2.1)	4.2 (2.4)
Commitment^b^	7.9 (1.4)	7.9 (1.7)

^a^Over the past 2 months since Time 1

^b^Potential range of scores = 1–9

### Measures

Participants completed a demographic questionnaire and a measure assessing use of monogamy maintenance efforts when interacting with a potential alternative partner, as well as whether attraction was reciprocated or not. They also completed measures of monogamy expectations, experiences with infidelity, and relationship commitment. Two validity items were embedded within the survey to identify unconscientious responders (e.g., “Pick the answer that starts with the letter B” with four response options). All measures have strong psychometric properties and were used successfully in the past.

#### Demographic Questionnaire

Participants completed a demographic measure designed for the current study that assessed a range of background information, including age, gender, sexual orientation, ethnicity, religion, educational level, relationship status, sexual experience, and current relationship duration.

#### Monogamy Maintenance Efforts and Reciprocation of Extradyadic Attraction

Participants completed the 24-item measure of monogamy maintenance efforts (Lee & O’Sullivan, 2018; see Appendix). They indicated whether they had engaged in any of the 24 efforts during an episode of strong attraction toward another member of the opposite sex during their current romantic relationship. The following prompt was provided: “We are interested in the behaviors that people perform to try to ensure that they maintain monogamy in their relationship with their current romantic partner. For each act, please indicate whether you have engaged in this behavior when you felt the most strongly drawn to, or experienced the greatest attraction to another member of the opposite sex who is not your partner.” Sample items include “turned down a plan that this other person tried to make with me” and “felt guilty that I flirted too much with this other person.” Higher values indicated greater efforts to maintain monogamy. This measure consists of three subscales: Proactive Avoidance (10 items), which includes efforts to avoid physical distance, face-to-face interactions, and conversational intimacies with a specific attractive other, or with members of the other sex more generally; Relationship Enhancement (7 items), which captures efforts to enrich one’s primary relationship sexually, materially, and emotionally to ensure monogamy is maintained; and Self-Monitoring and Derogation (7 items), which represents emotional and cognitive strategies in the face of extradyadic attraction, such as mentally downplaying one’s attraction to the attractive other and attempts to direct one’s attention back to the primary relationship (Lee & O'Sullivan, 2018). The internal consistency within the total monogamy maintenance (MM) inventory (*α* = .82) and its subscales (Proactive Avoidance *α* = .78, Relationship Enhancement *α* = .75, and Self-Monitoring and Derogation *α* = .76) were considered acceptable to good in the current sample. In addition, participants were asked whether the attraction was reciprocated or unreciprocated (experienced by the participant or the attractive alternative only).

#### Monogamy Expectations

Participants completed six items that assess expectations of sexual and romantic monogamy in general, in their current relationship, and perceptions of their partners’ expectations of sexual and romantic monogamy (Thompson & O’Sullivan, 2016b). Items were rated on a 5-point Likert scale, ranging from 1 (*Not at all*) to 5 (*Absolutely always*). An additional item assessed whether participants had established an explicit monogamy agreement with their primary partners (*yes*, *no*, and *not sure*).

#### Infidelity History

Participants indicated whether they had engaged in sexual and/or romantic infidelity during the course of their current romantic relationship. They were free to define the term infidelity, consistent with previous research (Watkins & Boon, 2016). Forced choice items assessed whether participants had engaged in romantic (*yes/no*) or sexual (*yes/no*) infidelity in their current relationship, whether their partner had engaged in romantic (*yes/no*) or sexual (*yes/no*) infidelity in their current relationship, and whether participants suspected that their partner had engaged in romantic (*yes/no*) or sexual (*yes/no*) infidelity in their current relationship (Thompson & O’Sullivan, 2016b).

#### Relationship Commitment

Participants completed 22 items from a revised version of the Investment Model Scale (Rusbult et al., 1998) with regard to their current primary romantic relationship, with subscales measuring relationship commitment, satisfaction, investment, and alternatives to the relationship. One item on the Investment Model Scale was revised to encompass romantic relationships beyond dating relationships (from “If I *weren’t dating* my partner, I would do fine—I would find another appealing person to date” to “If I *weren’t with* my partner, I would do fine—I would find another appealing person to date”). The IM Scale consists of seven global items examining relationship commitment that are rated on a 9-point Likert scale ranging from *Do not agree at all* (0) to *Agree completely* (8). Relationship satisfaction, relationship investment, and alternatives to the relationship were each measured with five items rated on a 9-point Likert scale ranging from *Do not agree at all* (0) to *Agree completely* (8). Higher values indicate greater levels of each dimension. Coefficient alphas for commitment (*α* = .85), satisfaction (*α* = .95), the quality of alternatives (*α* = .90), and investment (*α* = .82) were good to excellent in the current sample.

### Procedure

Those who met the eligibility criteria were directed to the informed consent form, then the online survey and debriefing form. Participants were compensated $2 USD via their MTurk^®^ accounts for their participation.

After 2 months, all prior respondents were invited to participate in the follow-up study via an advertisement posted on MTurk^®^ that could be viewed only by participants of the initial study. Respondents (*n* = 131) indicated whether they had the same partner as at the initial assessment. They then completed a subset of the original questionnaires with regard to the prior 2 months, including monogamy maintenance use, relationship commitment, and experiences with infidelity. Those who indicated that their relationships had dissolved during the interim period were not asked to report relationship commitment at follow-up. Participants were compensated monetarily ($1 US) via their MTurk^®^ accounts for their participation in the follow-up survey.

## Results

### Data Screening and Conditioning

Of the 350 participants recruited, 63 participants were excluded from the analyses because they did not complete the survey, did not meet the eligibility criteria, or were unconscientious or duplicate responders. The final sample consisted of 287 participants. Data were screened and conditioned using procedures outlined by Tabachnick and Fidell (2012). All data were missing completely at random and were replaced using estimation maximization (Little’s MCAR test *χ*^2^ = 1469.4, *df* = 1458, *p* = .412). No variable exceeded 5% in missing data.

A total of 131 participants (45.6% response rate) completed the follow-up survey 2 months after initial recruitment. The initial and the follow-up samples did not significantly differ on demographic variables (gender, relationship status, age, and ethnicity). Responders and non-responders also did not significantly differ on demographic variables. Data were screened for outliers and conditioned similarly to the initial data set.

Scores on relationship satisfaction, investment, and commitment were substantially significantly negatively skewed, in that a large percentage of participants were highly satisfied, invested, and committed to their relationships, consistent with the sample characteristics. The dependent variables (i.e., total monogamy maintenance efforts used and efforts used in each of the three factors) were significantly positively skewed, indicating that most individuals used a small number of efforts. We subsequently used nonparametric tests of significance and corrections for non-normality to analyze these data.

#### Descriptive Statistics

Two-thirds of participants (61.7%) reported experiencing an episode of extradyadic attraction in their current relationship. The majority (*n* = 177; 97.7%) of those who reported extradyadic attraction reported engaging in at least one monogamy maintenance strategy (*M *= 7.49; SD = 4.4; range = 0–20; see Table 2). Of these, some type of Proactive Avoidance was most common (90.4%; *M *= 3.6 efforts; SD = 2.5), followed by Self-Monitoring and Derogation (80.8%; *M *= 2.5 efforts; SD = 2.0) and Relationship Enhancement (61.0%; *M *= 1.4 efforts; SD = 1.7). In the total sample, 12.9% reported infidelity. Specifically, 8.4% of participants reported having engaged in romantic infidelity in their current relationships, 8% reported having engaged in sexual infidelity, and 3.5% reported having engaged in both romantic and sexual infidelity. Three of the four Investment Model variables were strongly negatively skewed, with the majority of respondents reporting high satisfaction (*M* = 7.3; SD = 1.6; range = 1.7–9), high investment (*M* = 7.4; SD = 1.4; range = 2.7–9), and high commitment (*M* = 7.9; SD = 1.4; range = 3.4–9). Log transformations of these variables were used in the relevant analyses. Perceived quality of alternatives (*M* = 3.8; SD = 2.1; range = 1–9) was normally distributed.

**Table 2:** Monogamy maintenance use in participants experiencing extradyadic attraction

Strategy used	Initial recruitment (*n* = 177)	Two-month follow-up (*n* = 67)
Monogamy maintenance (MM)	7.49 (4.4)	7.7 (4.4)
Proactive Avoidance	3.6 (2.5)	3.1 (2.5)
Self-Monitoring and Derogation	2.5 (2.0)	2.5 (1.8)
Relationship Enhancement	1.4 (1.7)	2.1 (1.7)
Any MM use	97.7%	98.5%
Proactive Avoidance use	90.4%	87.9%
Self-Monitoring and Derogation	80.8%	84.8%
Relationship Enhancement	61.0%	75.8%

In the follow-up data (*n* = 131), 67 participants (51.1%) reported experiencing extradyadic attraction in the prior 2 months. Most (98.5%) of these reported at least one monogamy maintenance strategy (*M* = 7.7; SD = 4.4; range = 0–19; see Table 2). Proactive Avoidance again was the most widely endorsed (87.9%), with a mean of 3.1 efforts (SD = 2.5), followed by Self-Monitoring and Derogation (84.8%; *M *= 2.5; SD = 1.8) and Relationship Enhancement (75.8%; *M *= 2.1; SD = 1.7). In the follow-up sample overall, 15.2% of participants reported engaging in infidelity in the prior 2 months. Romantic infidelity was more common (11.5%) than was sexual infidelity (9.2%), but 5.3% of participants reported engaging in both. A chi-square test for independence (with Yates’ continuity correction) indicated no significant difference between Study 2 and follow-up samples when comparing combined infidelity rates, *χ*^2^(1, *n* = 131) = .18, *p* = .67, *phi* = .06. Two chi-square goodness-of-fit tests indicated there was no significant difference in the proportion of individuals in the follow-up sample who reported engaging in romantic infidelity, *χ*^2^(1, *n* = 131) = 1.58, *p* = .21, or sexual infidelity, *χ*^2^(1, *n* = 131) = .24, *p* = .62, as compared to the Study 2 sample. Six participants (4.6%) reported that their romantic relationship from the initial time point had dissolved over the past 2 months. Satisfaction, investment, and commitment were again strongly negatively skewed, with the majority of respondents reporting high satisfaction (*M* = 7.3; SD = 1.7; range = 1–9), high investment (*M* = 7.5; SD = 1.2; range = 4.1–9), and high commitment (*M* = 7.9; SD = 1.7; range = 3.4–9). As before, log transformations of these variables were used in the relevant analyses. Perceived quality of alternatives (*M* = 4.2; SD = 2.4; range = 1–9) was normally distributed.

### Preliminary Analyses

A Mann–Whitney *U* test was conducted to assess gender differences in the use of monogamy maintenance (MM) efforts overall and in the use of the three MM subtypes. No significant differences were found in men and women’s uses of MM overall (*U* = 10134.0; *z* = − .23, *p* = .82), Proactive Avoidance (*U* = 10198.5; *z* = − .14, *p* = .88), Relationship Enhancement (*U* = 9376.5; *z* = − 1.38, *p* = .17), and Self-Monitoring and Derogation (*U* = 10013.5; *z* = − .41, *p* = .68). A Mann–Whitney *U* test assessed associations between relationship status and MM use. A statistically significant difference in the number of Self-Monitoring and Derogation efforts was found between the two different groups (Grp1, married/cohabiting, *n* = 156; Grp2, dating relationship, *n* = 131), *U* = 8679.5, *z* = − 2.3, *p* = .02. A subsequent examination of median differences did not reveal any differences between the groups (*Md*s for Grp1 and Grp2 = 1.0). No other significant associations were found between relationship status and strategy use. Kruskal–Wallis H tests were conducted to examine associations between having a monogamy agreement with one’s partner (*yes, no,* and *not sure*) and monogamy maintenance efforts (total MM, Proactive Avoidance, Relationship Enhancement, and Self-Monitoring and Derogation). No significant differences were found between the three groups in any type of efforts used. Overall, gender, relationship status, and monogamy agreement status were not associated with monogamy maintenance.

Post hoc analyses were conducted to explore the relationships between monogamy maintenance use with age and with relationship length. The relationship between age (in years) and MM use was investigated using Spearman’s rho, as MM use was not normally distributed. There was a small, negative correlation between age and Relationship Enhancement, *r*_*s*_ = – .15, *n* = 280, *p* < .05, with higher age associated with lower levels of Relationship Enhancement efforts. Age was not significantly associated with the other two MM subtypes, nor with overall MM use. Spearman’s rho was used to examine the relationship between relationship length (in months) and MM use. A small, negative correlation was identified between relationship length and Relationship Enhancement, *r*_*s*_ = – .15, *n* = 276, *p* < .05; longer relationship duration was associated with lower levels of Relationship Enhancement efforts. A small, positive correlation was identified between relationship length and Self-Monitoring and Derogation, *r*_*s*_ = .14, *n* = 276, *p* < .05, with longer relationship duration associated with greater endorsement of Self-Monitoring and Derogation.

### Associations Between Relationship Commitment and Monogamy Maintenance Types

**Fig. 1:**
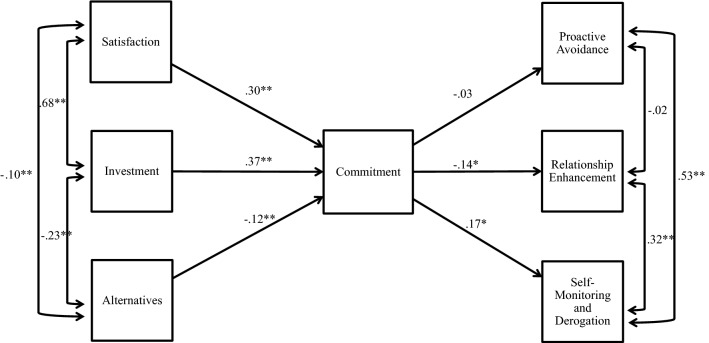
Visual representation of the associations between Investment Model and monogamy maintenance efforts

Structural equation modelling was conducted using the lavaan package in R^®^ software to examine the association between the Investment Model and monogamy maintenance strategy use in episodes of extradyadic attraction (see Table 3). The Yuan–Bentler correction was applied to account for non-normally distributed data in a sample of this size. First, the adequacy of model fit between the Investment Model Scale variables and three Monogamy Maintenance Inventory subscales was examined. Following recommendations by Tabachnick and Fidell (2012), the model fit was assessed via robust comparative fit index, robust root-mean-square error of approximation (RMSEA), and the standardized root-mean-square residual (SRMR) and was found to be acceptable to good, *χ*^2^ = 475.57, *df* = 21; robust CFI = .97; SRMR = .04; RMSEA = .08. A visual representation of the associations between variable sets is depicted in Fig. 1. Relationship satisfaction (*r* = .30, *n* = 287, *p* < .001), investment (*r* = .37, *n* = 287, *p* < .001), and alternatives (*r* = − .12, *n* = 287, *p* < .001) were significantly associated with relationship commitment in the expected directions, replicating the expected model (H1a). Relationship commitment predicted lower levels of Relationship Enhancement use (*r* = − .14, *n* = 287, *p* < .05), contrary to H1b, higher levels of Self-Monitoring and Derogation use (*r* = .17, *n* = 287, *p* < .05), and was unrelated to Proactive Avoidance, *r* = − .03, *n* = 287, *p* = .76.

**Table 3:** Model fit statistics for structural equation models

Description	Chi-square (*df*)	SRMR	RMSEA	Robust CFI
Investment model with monogamy maintenance	475.57 (21)***	.04	.08	.97
Monogamy maintenance predicting infidelity at follow-up	109.63 (21)***	.03	.05	.99

*N* = 287

*SRMR* standardized root mean residual, *RMSEA* root-mean-square error approximation, *TLI* Tucker–Lewis index, *CFI* comparative fit index

***p < .001

### Monogamy Maintenance as a Predictor of Infidelity at 2-Month Follow-up

Structural equation modelling was conducted to examine the ability of monogamy maintenance at Time 1 to predict reported infidelity outcomes at 2-month follow-up (see Table 3). Proactive Avoidance, Relationship Enhancement, Self-Monitoring and Derogation, and relationship commitment at T1 were regressed onto the romantic infidelity and sexual infidelity outcomes at T2, which were then regressed onto relationship commitment at T2. Self-Monitoring and Derogation at T1 was allowed to covary with the other two monogamy maintenance factors to reflect the correlations previously found (Lee & O'Sullivan, 2018). Romantic and sexual infidelity outcomes at T2 also were allowed to covary, reflecting the overlap between infidelity types. Commitment at T1 was regressed onto commitment at T2 to control for its effects. The Huber–White robust standard errors correction was applied to maximum likelihood estimation to account for non-normally distributed data in a sample of this size. The model converged after 85 iterations, *χ*^2^ = 109.63, *df* = 21. The model fit was again assessed via robust comparative fit index, robust root-mean-square error of approximation (RMSEA), and the standardized root-mean-square residual (SRMR) and was found to be good, robust CFI = .99; SRMR = .03; RMSEA = .05. However, the use of monogamy maintenance efforts at T1 was not significantly associated with romantic and sexual infidelity outcomes at T2 (H2a). As predicted, sexual infidelity at T2 was negatively associated with relationship commitment at T2, *r* = − .33, *n* = 119, *p* < .05, replicating previous findings (H2b); the same negative associations were not found between romantic infidelity and relationship commitment at T2.

### Associations Between Attraction Context and Monogamy Maintenance

A Kruskal–Wallis H test was conducted to examine monogamy maintenance use across four forms of extradyadic attraction (see Table 4). These forms were as follows: unreciprocated attraction of participant (Grp1); unreciprocated attraction of attractive alternative (Grp2); reciprocated attraction (Grp3); and reciprocated attraction unknown (Grp4). A significant difference was found across the four groups (Grp1, participant attraction, *n* = 38, Grp2, attraction of attractive alternative, *n* = 22, Grp3, reciprocated attraction, *n* = 78, Grp4, unknown, *n* = 33) in the total use of MM, and specifically in Proactive Avoidance and Self-Monitoring and Derogation. Two post hoc between-group comparisons were made using Mann–Whitney *U* tests, and a Bonferroni adjustment was applied to account for increased potential for type I error (*p* < .025). Significant small- to medium-sized group differences were found between participants experiencing unreciprocated and reciprocated attractions (Grp1 and Grp3) in the use of Proactive Avoidance, *U* = 1061.0, *z* = − 2.49, *p* = .01, *r* = .23, Self-Monitoring and Derogation, *U* = 964.5, *z* = − 3.08, *p* < .01, *r* = .29, and total MM, *U* = 955.5, *z* = − 3.11, *p* < .01, *r* = .29. Those who reported that the attraction was reciprocated used significantly more monogamy maintenance efforts as compared to those who reported unreciprocated attraction. In contrast, group differences were not found between the two unreciprocated attraction groups (Grp1 and Grp2) in the uses of Proactive Avoidance, *U* = 398.5, *z* = − .31, *p* = .76, Self-Monitoring and Derogation, *U* = 405.5, *z* = − .20, *p* = .84, or total MM, *U* = 403.0, *z* = − .23, *p* = .82.

**Table 4:** Use of monogamy maintenance by context of extradyadic attraction

Context	Monogamy maintenance strategy								
		Proactive Avoidance		Relationship Enhancement		Self-Monitoring and Derogation		Total	
	*N*	Md	*χ* ^2^	Md	*χ* ^2^	Md	*χ* ^2^	Md	*χ* ^2^
Participant attraction^a^	38	2		1		1		5.5	
Extradyadic partner attraction^b^	22	3		.5		1		5	
Reciprocated attraction^ab^	78	4		1		3		8	
Reciprocated attraction—unknown	33	4		1		2		8	
Kruskal–Wallis *χ*^2^			8.20*		.85		14.84**		13.49**

*N* = 171

^a^Post hoc Mann–Whitney *U* tests indicated significant group differences in uses of Proactive Avoidance, *U* = 1061.0, *z* = − 2.49, *p* = .01, Self-Monitoring and Derogation, *U* = 964.5, *z* = − 3.08, *p* < .01, and total MMI, *U* = 955.5, *z* = − 3.11, *p* < .01

^b^Post hoc Mann–Whitney *U* tests indicated significant group differences in uses of Self-Monitoring and Derogation, *U* = 548.0, *z* = − 2.61, *p* < .01, and total MMI, *U* = 541.5, *z* = − 2.64, *p* < .01, *p < .05, **p < .01

## Discussion

The main purpose of the current study was threefold: to provide an initial investigation into how relationship commitment influences how individuals manage their attraction to others when in a monogamous relationship, to identify whether these attempts were effective in maintaining monogamy, and to examine attraction contexts that incur monogamy maintenance efforts. Monogamy maintenance efforts are deliberate attempts by individuals to maintain monogamy in their relationships when facing temptations away from their primary relationship via extradyadic attraction. Insights into how individuals respond to common relationship threats posed by attractive others and their effectiveness ultimately may help individuals to focus on more effective means of maintaining monogamous relationships and reducing vulnerability to infidelity, which is viewed as a serious violation of trust and is a common precursor to relationship distress and breakup (Amato & Previti, 2003; DeMaris, 2013). Better understanding of monogamy maintenance and its limitations may contribute significantly to interventions aimed at developing behavioral efforts to strengthen monogamous relationships.

### Relationship Characteristics Associated with Monogamy Maintenance

Adults in monogamous relationships vary in their use of monogamy maintenance depending on their levels of relationship commitment. The multidimensional nature of monogamy maintenance was supported by the finding of differential patterns of use across relationship commitment levels. However, contrary to our hypothesis, Self-Monitoring and Derogation was the only factor that was positively associated with relationship commitment. Our findings indicate that individuals who were more committed to their relationships were more likely to perceive their extradyadic attraction as a threat and respond with self-directed behaviors, such as attempts to manipulate one’s emotions and derogating the attractive other. This finding is consistent with prior research establishing the derogation effect (Lydon et al., 1999, 2003) and suggests that the derogation effect can extend beyond automatic, implicit processes. Although the identities of the attractive alternative were not identified by participants in the current study, monogamy maintenance encompasses intentional behavioral efforts that may target non-fleeting extradyadic attraction. In comparison with the other two types of monogamy maintenance, Self-Monitoring and Derogation appears to be more reactive and less proactive in nature, aimed at cajoling one’s attention back to the primary relationship when it has already been drawn toward the attractive alternative (e.g., “reminded myself the importance of being faithful,” “told myself that that this other person was bad for me”). The reactive nature of Self-Monitoring and Derogation efforts may indicate that individuals who are highly committed to their relationships do not expect to experience extradyadic attraction and may not respond to the attraction until it is well developed and more obviously a threat.

In comparison with Self-Monitoring and Derogation, Relationship Enhancement was negatively associated with relationship commitment, which was contrary to our hypothesis, suggesting that individuals who were more committed to their relationships were less likely to report working on improving the quality of the relationship as a method of avoiding extradyadic involvement. Individuals may perceive their efforts to enhance their relationship as an end to itself, as compared to a means by which to protect their relationships. It also may be that the more committed an individual is to a relationship, the less effort is made to work on one’s relationship even in the face of a potential threat—taking it for granted in a sense. In addition, the items on the Relationship Enhancement subscale may reflect the processes in which individuals engage to enhance or deepen a new relationship, reflecting the process of courtship (e.g., “Had a physical relationship with my partner to deepen our bond”). These behaviors may ultimately reflect a constellation of common courtship behaviors used to establish a monogamous relationship. This explanation also is supported by the findings that increased age and relationship length were weakly correlated with lower levels of Relationship Enhancement. Lastly, Proactive Avoidance was not associated with relationship commitment, indicating that these efforts are commonly used regardless of how committed an individual is to their relationship. The aim of Proactive Avoidance strategies is to restrict opportunities to interact with attractive alternative partners as a way to inhibit the development of intimacy with attractive others. Individuals may find themselves constrained by social norms and overlapping social circles in their attempts to avoid interacting with attractive others who may be encountered at work, social, or leisure activities.

Our findings support the use of the Investment Model as a theoretical framework in predicting monogamy maintenance. Prior research has used this framework to predict infidelity (Drigotas et al., 1999; Martins et al., 2016), conflict resolution (Guerrero & Bachman, 2008), and willingness to sacrifice in intimate relationships (Van Lange et al., 1997). The four components of the Investment Model (commitment, satisfaction, investments, and perceived quality of alternatives) predicted one another as expected, replicating previous research (Guerrero & Bachman, 2008; Le & Agnew, 2003; Martins et al., 2016), which then was associated with a novel relationship maintenance outcome, in this case, monogamy maintenance.

Of note, no differences in use of monogamy maintenance emerged for gender, relationship status, or whether a couple had an explicit monogamy agreement in place. Thus, monogamy maintenance appears to be a widely adopted set of behaviors that the majority of individuals in relationships employ in response to extradyadic attraction. The lack of association between relationship status and monogamy maintenance suggests that “structural” commitment, represented by relationship status (married/cohabiting or dating), did not influence monogamy maintenance use as much as “attitudinal” commitment, represented by self-rated levels of commitment (Lydon et al., 1999) and by relationship length. The finding that having an explicit monogamy agreement in place was unrelated to monogamy maintenance use suggests that individuals have internalized norms about maintaining exclusivity.

### Monogamy Maintenance and Monogamy Success

Use of some form of monogamy maintenance did not predict later success in resisting romantic or sexual infidelity, contrary to hypotheses. Monogamy maintenance efforts were identified by respondents as their attempts to realign interest in a primary partner and avoid an attractive other, but these efforts did not appear to be effective in thwarting interest in an attractive other. Monogamy maintenance previously was found to be predictive of extradyadic flirtation (Lee & O'Sullivan, 2018). Self-Monitoring and Derogation efforts were found to be positively associated with flirtation, whereas Relationship Enhancement efforts were negatively associated with flirtation (Lee & O'Sullivan, 2018), in patterns consistent with the current findings. Monogamy maintenance appears to be more effective in redirecting individuals from engaging in flirtation, a subtler and more socially tolerated extradyadic behavior which may lead to infidelity, but other motivations and risk factors likely override monogamy maintenance when sexual or romantic infidelity is being considered. It may be that that efforts to maintain monogamy were impeded by other characteristics, such as sociosexuality (Feldman & Cauffman, 1999), attachment style (Beaulieu-Pelletier, Philippe, Lecours, & Couture, 2011), or impulsivity (McAlister, Pachana, & Jackson, 2005), that place individuals at higher risk of extradyadic involvement. In addition, we did not assess the behavior of the attractive other. Those individuals may have been especially persistent upon realizing that attraction was reciprocated and difficult to avoid ultimately, given that most extradyadic partners are individuals well integrated into one’s life prior to involvement.

Moreover, the number of different monogamy maintenance efforts used and infidelity outcomes were unrelated. Although used as a measure of increased effort, more varied monogamy maintenance use might not actually translate into greater effectiveness, as the number of strategies used likely includes both successful and unsuccessful efforts. We did not assess the intensity and frequency of MM use. For example, it may be that one strategy, used consistently, is more effective in maintaining monogamy than using a number of different strategies inconsistently. Overall, even when the intent is there, the number of monogamy maintenance efforts used did not effectively protect monogamy, at least in our sample.

While holding constant initial levels of relationship commitment, individuals who engaged in an extradyadic sexual relationship over the course of two months reported lower relationship commitment at follow-up, consistent with prior findings that infidelity is associated with relationship disruption and breakup (Allen & Atkins, 2012; DeMaris, 2013; Drigotas et al., 1999). Surprisingly, extradyadic romantic involvement was not associated with lower relationship commitment in the current study, contrary to our hypotheses based upon prior research linking emotional infidelity to relationship and personal distress (Carpenter, 2012; Drigotas et al., 1999; Leeker & Carlozzi, 2014). Individuals may find themselves seeking emotional intimacy and support from alternative partners, friendships, and other relationships, while remaining committed to continuing their primary relationships. Qualitative research has uncovered the multifaceted nature of monogamy, and also how one dimension of monogamy, such as sexual exclusivity, may be valued over another, such as emotional exclusivity, in a committed relationship (Anderson, 2010). Our counterintuitive findings highlight the need to examine romantic infidelity as a phenomenon independent of its co-occurrence with sexual infidelity, and to explore its correlates, outcomes, and motivations.

### Monogamy Maintenance as a Response to Relationship Threat

Although most individuals in our sample reported some use of monogamy maintenance efforts, individuals who experienced an episode of reciprocated attraction with an attractive other used the highest variety of efforts, consistent with our hypothesis about greater perceived threat to monogamy posed by reciprocated attraction. Those attracted to individuals within their social circles may experience a tension between efforts to maintain monogamy and social propriety. For example, total avoidance of a work colleague to whom one may be attracted is often not feasible or tolerated, and derogating the attractiveness of a family friend may be socially inappropriate.

Those experiencing reciprocated attraction reported twice as many Proactive Avoidance and Self-Monitoring and Derogation efforts than did those experiencing unreciprocated attraction. These types of strategies represent efforts aimed at building physical and emotional distance from a potential alternative partner and redirecting one’s own attention back to the primary relationship. Overall, those experiencing episodes of extradyadic attraction perceived to confer greater threat to monogamy appeared to engage in greater levels of proactive and reactive efforts, aimed at both the attractive other and oneself.

### Limitations and Future Directions

A number of limitations must be noted with regard to the current study. Our sample was limited to heterosexual U.S. residents who were active users of online technology. Future research would benefit from specific explorations of monogamy maintenance among sexual minorities, to examine how differences previously found in attitudes toward and expectations for monogamy between heterosexual, gay/lesbian, and bisexual individuals in intimate relationships may be reflected in monogamy maintenance efforts (Hoff & Beougher, 2008; Hosking, 2013; Mark et al., 2014), and sampling of non-U.S. participants or those recruited by other means than online.

Similar to all survey research, our examinations are limited by the potential for self-selection, and biased or inaccurate recall. In particular, the strong social norm for monogamy may have increased self-enhancing response patterns regarding extradyadic involvement. We ensured anonymity to aid in the reporting of socially sensitive information. The rates of infidelity in our sample were relatively low, yet they were consistent with those from prior research (9.1%; Watkins & Boon, 2016), supporting the validity of our findings. Furthermore, the use of a short-term follow-up likely facilitated more accurate recall of behaviors over a specific and shorter time frame. Approximately half (45.6%) of our original sample completed the follow-up survey. Although responders did not differ from non-responders in terms of demographic variables, it is possible that self-selection bias may have excluded certain participants (e.g., those who experienced infidelity) from our follow-up sample. Results should be interpreted in light of this caution.

The small associations found between age and relationship length with monogamy maintenance efforts were unexpected and not predicted, to our knowledge, by findings in the existing literature. Given that the current study was exploratory in nature and focused on identifying associations between monogamy maintenance and relationship commitment, we could not explore these associations in greater detail. These findings suggest that many relationship characteristics inform the practices of monogamy, and possibly cohort effects are at play. We welcome future confirmatory and theory-driven explorations into these variables and their associations with monogamy maintenance.

Our finding that greater perceived threat to one’s monogamy appears to trigger stronger use of monogamy maintenance replicates prior findings on derogation of attractive alternatives (Lydon et al., 1999, 2003) and extends such findings to effortful behavioral responses to threats to monogamy. The field would benefit from further research on the contexts of extradyadic attraction that confer the most threat to monogamous relationships and trigger the strongest protective responses, exploring factors such as duration of attraction, anonymity, relationship intimacy, frequency of contact, and degree of social network overlap. In particular, the range of motivations for avoiding the attractive alternative was not assessed in the current study. It also is unclear whether respondents experienced conflicting approach motivations that countered those motivating them to avoid the attractive other. Qualitative research using interviews may provide insights into the thought processes, motivations, and emotional reactions that individuals experience during an episode of extradyadic attraction, including negative forms such as cognitive dissonance and regret, as well as positive forms such as increases in self-esteem and sexual arousal. Such research could help to explore which efforts or combination of efforts is ultimately most successful or unsuccessful in maintaining monogamy.

### Conclusions and Implications

The current study provides an initial examination of the use and efficacy of individuals’ attempts to protect their monogamous relationships from attractive alternatives. As its efficacy in deterring infidelity threat is questionable, monogamy maintenance use may be more usefully conceptualized as warning signs against relationship threat, or attempts to refocus attention on one’s primary partner. Further quantitative and qualitative examinations may help to identify the most effective monogamy maintenance strategies and the ways in which individuals engage in them successfully in service of relationship longevity. Monogamy is widely expected and adopted implicitly by many in intimate relationships, yet individuals commonly face attractive alternatives, revealing a gap in the desire and practices to be consistent with monogamy ideals. Educators and therapists should explore the meaning of monogamy to couples and identify which components are most important, while destigmatizing extradyadic attraction to facilitate discussions about monogamy maintenance and threat identification. Ultimately, this study aims to spur further explorations regarding the practices of monogamy and the agentic role that individuals play in improving intimate relationship quality and maintenance.

## Appendix: Monogamy Maintenance Inventory

People in relationships often find themselves being drawn toward or attracted to someone who is not their primary partner. Please recall an episode during your current relationship where you have felt the most strongly drawn to, or experienced the greatest attraction to another member of the opposite sex who is not your primary partner.

On the following pages are a series of acts or behaviors. In this study, we are interested in the behaviors that people perform to try to ensure that they maintain monogamy in their relationship with their current romantic partner. For each act, please indicate whether you have engaged in this behavior when you felt the most strongly drawn to, or experienced the greatest attraction to another member of the opposite sex who is not your partner:

1. Avoided spending time with this other person
2. Turned down spending time with this other person
3. Avoided being alone with this other person
4. Distanced myself from this other person
5. Turned down a plan that this other person tried to make with me
6. Avoided having close relationships with members of the opposite sex (outside of family)
7. Deleted their phone number
8. Removed them from my social media accounts (like Facebook, Instagram, Snapchat, etc.)
9. Avoided finding out more about this other person
10. Avoided getting to know this other person better to avoid developing a “crush” on them
11. Had a physical relationship with my partner to deepen our bond
12. Took my partner out on a date
13. Bought my partner a gift
14. Engaged in sexual acts with my partner
15. Made sure that I looked nice for my partner
16. Told my partner how important they are to me
17. Made myself “extra attractive” for my partner
18. Felt guilty that I flirted too much with this other person
19. Reminded myself the importance of being faithful
20. Told myself that this other person was bad for me
21. Told myself about the potential negative consequences of cheating on my partner
22. Told myself that I needed to commit to my partner
23. Looked for unflattering things in this other person
24. Told myself that I was dependent on my partner

Researchers can calculate one overall measure of monogamy maintenance by taking the mean of all the items from the scale. Alternatively, researchers can calculate the following monogamy maintenance subscales by taking a mean of the items in the subscale:

Proactive Avoidance: Items 1 through 10

Relationship Enhancement: Items 11 through 17

Self-Monitoring and Derogation: Items 18 through 24
